# Discriminant analysis of principal components and pedigree assessment of genetic diversity and population structure in a tetraploid potato panel using SNPs

**DOI:** 10.1371/journal.pone.0194398

**Published:** 2018-03-16

**Authors:** Sofía. I. Deperi, Martín. E. Tagliotti, M. Cecilia Bedogni, Norma C. Manrique-Carpintero, Joseph Coombs, Ruofang Zhang, David Douches, Marcelo. A. Huarte

**Affiliations:** 1 National Research Council of Argentina (CONICET), Ciudad Autónoma de Buenos Aires, Buenos Aires, Argentina; 2 National Institute for Agricultural Technology (INTA), EEA INTA Balcarce, Balcarce, Buenos Aires, Argentina; 3 Michigan State University, East Lansing, Michigan, United States; 4 Inner Mongolia University, Hohhot, Inner Mongolia, China; Agriculture and Agri-Food Canada, CANADA

## Abstract

The reported narrow genetic base of cultivated potato (*Solanum tuberosum*) can be expanded by the introgression of many related species with large genetic diversity. The analysis of the genetic structure of a potato population is important to broaden the genetic base of breeding programs by the identification of different genetic pools. A panel composed by 231 diverse genotypes was characterized using single nucleotide polymorphism (SNP) markers of the Illumina Infinium Potato SNP Array V2 to identify population structure and assess genetic diversity using discriminant analysis of principal components (DAPC) and pedigree analysis. Results revealed the presence of five clusters within the populations differentiated principally by ploidy, taxonomy, origin and breeding program. The information obtained in this work could be readily used as a guide for parental introduction in new breeding programs that want to maximize variability by combination of contrasting variability sources such as those presented here.

## Introduction

Modern potato cultivars were developed from a few clones brought from the American continent to Europe in the XVI century. After the Irish potato famine in XIX, its genetic base was drastically narrowed [1]. To recover genetic diversity, cross-breeding of potato and systematic utilization of related wild species has been made after the late blight epidemics [2]. These species have great variability for many desirable agronomic characters [1, 3–7]. However, many studies suggest that potato genetic base is still narrow [8; 9]

The genetic characterization of available potato populations allows us to assess their diversity and structure and to identify the genotypes that could work as a source of new alleles in potato breeding programs. The aim of these programs has been to incorporate resistance to biotic and abiotic stresses, as well as to develop better processing qualities and combine these characters with high yield and commercial quality [10], using conventional or molecular techniques. More specifically, molecular markers have been used to evaluate the genetic diversity of potatoes in many populations [11–15].

The genetic structure of a population can be assessed by several methods using molecular markers information, such as STRUCTURE [16], EIGENSTRAT [17], kinship [18], discriminant analysis of principal components (DAPC) [19], methods based on genetic distances [20–21], among others. Particularly, DAPC analysis is a multivariate method used to identify and describe clusters of genetically related individuals. Genetic variation is partitioned into two components: variation between groups and within groups, and it maximizes the former. Linear discriminants are linear combinations of alleles which best separate the clusters. Alleles that most contribute to this discrimination are therefore those that are the most markedly different across groups. The contributions of alleles to the groupings identified by DAPC can allow identifying regions of the genome driving the genetic divergence among groups [19].

Information generated from genealogy in breeding programs is generally not used for the assessment of genetic diversity and characterization of populations. However, it is very valuable as it complements the data provided by the molecular markers and helps to understand the genetic base and history of a population.

The objective of this work was to provide a methodology based on a molecular characterization using DAPC and a pedigree characterization to assess the genetic structure and diversity of a potato population made of genotypes of diverse origins.

## Materials and methods

### Plant material

The population consisted of 231 genotypes from the germplasm collection of EEA INTA Balcarce potato breeding program, selected to represent different genetic sources of diversity. It contains potato varieties from China, Uruguay, Chile, Peru, United States, Netherlands, Brazil, Bolivia, United Kingdom, Argentina and advanced clones from the breeding programs of the International Potato Center (CIP Perú) and of the National Institute of Agricultural Technology (INTA Balcarce). It also included some varieties from Group Andigena (*Solanum tuberosum* Gp. Andigena), wild species (*Solanum chacoense* Bitt. and *Solanum tarijense* Hawkes (syn *Solanum berthaultii*)), and a *Solanum tuberosum* var. Calén x *Solanum gourlayii* hybrid and their reciprocal (S1 Table).

### DNA extraction

DNA was extracted from 100 mg of fresh young leaf tissue of each genotype grown under greenhouse conditions or under field conditions. The QIAGEN Plant mini kit ® (Qiagen, Valencia, CA, USA) was used for the extraction. DNA quality and quantity of each sample was determined with electrophoresis in 1% (w/v) agarose gels and spectrophotometry. Final DNA concentration was determined using PicoGreen quantitation (Eugene, Oregon).

### Molecular markers

The population was genotyped with the Illumina Infinium Potato SNP Array V2 (12,808 SNPs, including the markers from SolCAP Infinium 8303 Potato SNP Array) [22–23]. Illumina GenomeStudio software (Illumina, San Diego, CA) was used for initial sample quality assessment. Tetraploid (5-cluster AAAA, AAAB, AABB, ABBB, BBBB) genotyping was based on theta values, using a custom script from the SolCAP project reported in [14]. Following 5-cluster genotype calling and filtering, minor allele frequencies (MAF) was calculated using *adegenet* package (1.4–2 version) [24] for R software (3.4.0 version) [25], and only genotypes with MAF greater than 0.05 were retained. Finally, 4,859 high quality markers were used for analysis.

### Population structure and genetic diversity

The population structure was analyzed by a DAPC [20] using the *adegenet* package [24] for R software [25]. The *find*.*clusters* function was used to detect the number of clusters in the population. It uses K-means clustering which decomposes the total variance of a variable into between-group and within-group components. The best number of subpopulations has the lowest associated Bayesian Information Criterion (BIC). A cross validation function (*Xval*.*dapc*) was used to confirm the correct number of PC to be retained. In this analysis, the data is divided into two sets: a training set (90% of the data) and a validation set (10% of the data) The member of each group are selected by stratified random sampling, which ensures that at least one member of each group or population in the original data is represented in both training and validation sets. DAPC is carried out on the training set with variable numbers of PCs retained, and the degree to which the analysis is able to accurately predict the group membership of excluded individuals (those in the validation set) is used to identify the optimal number of PCs to retain. At each level of PC retention, the sampling and DAPC procedures are repeated many times [26]. The best number of PCs that should be retained is associated with the lowest root mean square error. The resultant clusters were plotted in a scatterplot of the first and second linear discriminants of DAPC. The analysis was repeated into the largest groups to detect if they were also structured. SNPZIP analysis (*adegenet*) uses DAPC analysis to identify alleles with the largest contributions to form the linear discriminants and assign the genotypes to the clusters, in order to know the population structure. To confirm the allocation of individuals to clusters by DAPC analysis, a Nei genetic distance matrix [27] was calculated with the StAMMP package of R software using the SNPs information. Then the resulting matrix was plotted as a dendrogram using the ward method with the InfoStat software [28].

The analysis of molecular variance (AMOVA), the coefficient of genetic differentiation among populations (F_st_) and a subpopulation inbreeding coefficient (F_is_) were calculated using GenAlEx software (6.5 version) [29] with 999 permutations. Expected and observed heterozygosity (H_e_, H_o_) and Percentage of polymorphic loci were also calculated using GenAlEx software.

### Pedigree information

A database of pedigree information of the potato lines and varieties was compiled and presented in a tree using *Peditree* ® software [30]. Information was taken from INTA Balcarce potato breeding program, CIP breeding program and from the *Potato pedigree database* (Wageningen) public database [31]. The number of generations considered was the maximum available for each clone. Inbreeding and co-ancestry coefficients were calculated using *Peditree* ® software and were used to calculate the Coefficient of Relationship (CR) [32] among the individuals of each group.

Maximum average contribution (MAC) was calculated as the frequency of the contribution of each parent to each offspring according to the level in the genealogy, in relation to the frequency of occurrence of each parent in the genotypes that make up each subpopulation. A maximum of five generations of ancestors was used for this analysis.

## Results

### Population structure and genetic diversity

**Fig 1:**
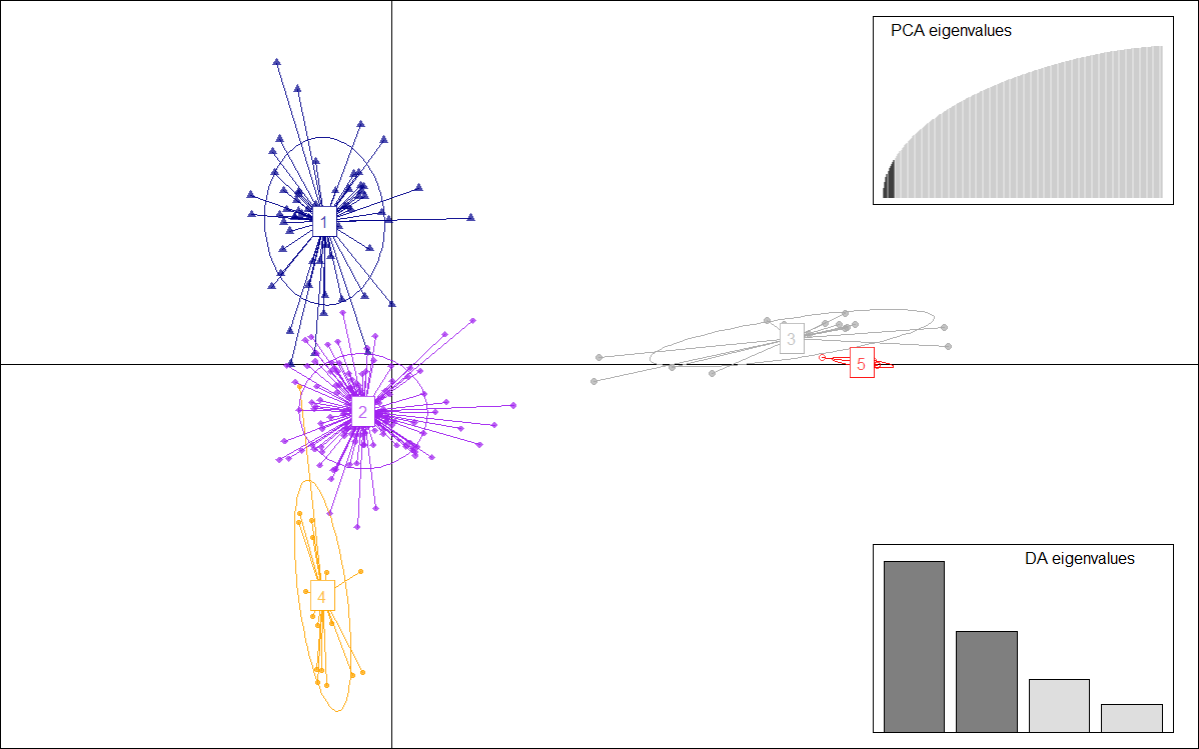
Discriminant analysis of principal components (DAPC) for 231 genotypes of the germplasm collection of INTA Balcarce potato breeding program. The axes represent the first two Linear Discriminants (LD). Each circle represents a cluster and each dot represents an individual. Numbers represent the different subpopulations identified by DAPC analysis.

The number of detected clusters was five, in coincidence with the lowest BIC value using *find*.*clusters* function (Fig A in S1 Fig). DAPC analysis was carried out using the detected number of clusters (Fig 1 and S2 Table). Ten first PCs (24,7% of variance conserved) of PCA and four discriminant eigenvalues were retained. These values were confirmed by a cross validation analysis (Fig B in S1 Fig). Membership coefficients of the genotypes to each group were between 0.721 and 1, thus confirming that there was low admixture and that the population was structured. Exceptions to these values were clone B 99.558.1, clone CIP393595.1 and Kennebec whose values were 0.620, 0.616 and 0.543 respectively.

**Fig 2:**
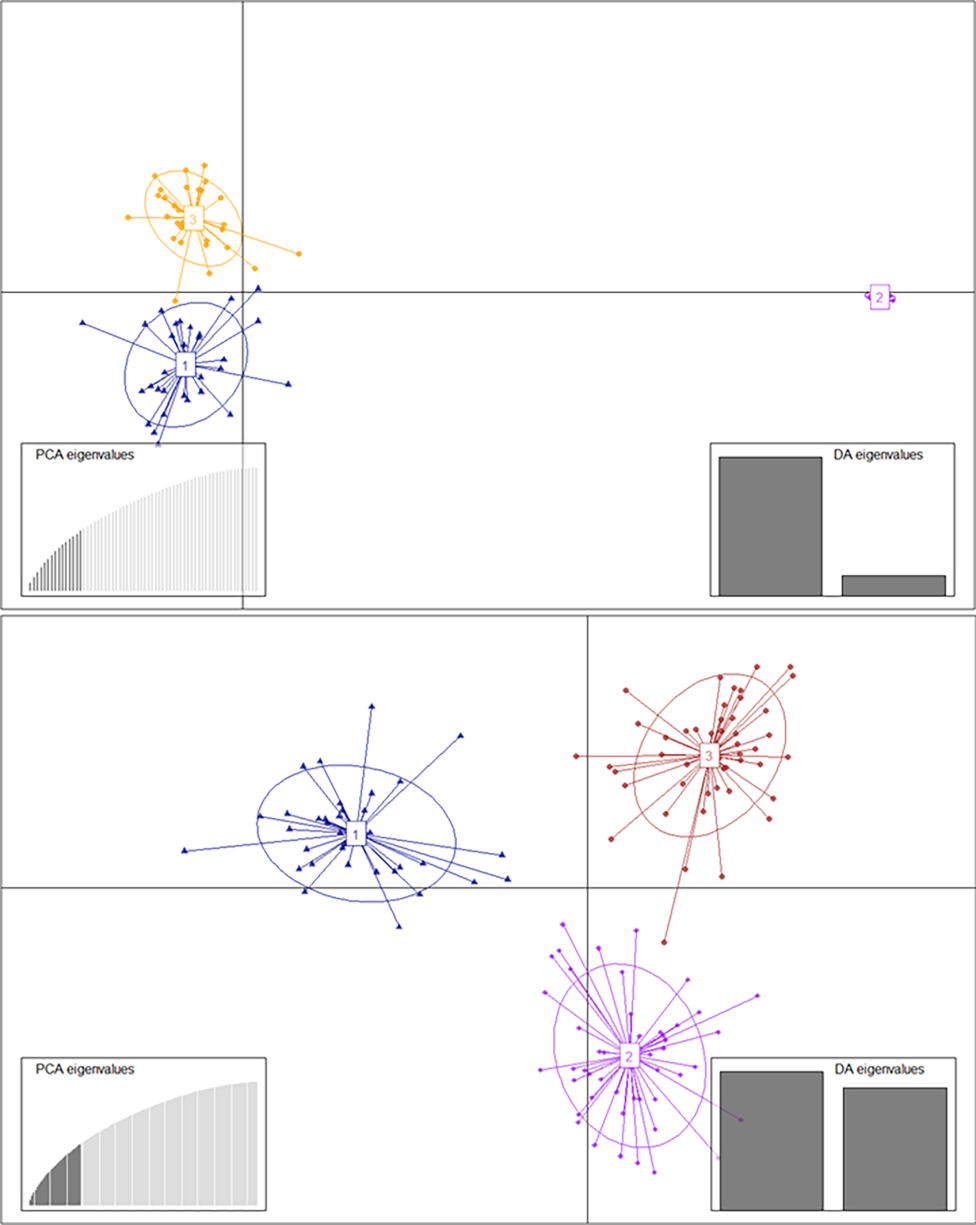
Discriminant analysis of principal components (DAPC) for 231 genotypes of the germplasm collection of INTA Balcarce potato breeding program. (a) subpopulation 1 (b) subpopulation 2. The axes represent the first two linear discriminants (LD). Circles represent groups and dots represent individuals. Numbers represent the different subpopulations identified by DAPC analysis.

In Fig 1, Linear Discriminant 1 (LD 1) separated among the *Solanum* species (Groups 1, 2 and 4 = *S*. *tuberosum* Gp. Tuberosum; Group 3 = *S*. *tuberosum* Gp. Andigena; Group 5 = *S*. *chacoense* and *S*. *tarijense*, diploid species), and Linear Discriminant 2 (LD 2) separated among *S*. *tuberosum* groups (1, 2 and 4). Groups 2, 3 and 5 were roughly at the same level with respect to LD 2, and groups 1 and 4 were above and below them, respectively. DAPC analysis of subpopulations 1 and 2, separated each of them into three groups (S3 Table and Fig 2). However, in subpopulation 1 (Fig 2A), one of the subgroups is more distant from the others with respect to LD1. The coefficients of clones in Subpopulation 1 were between 0,764 and 1 but the exceptions were genotypes Araucana INTA and B 07.537.4 with 0.666 and 0.644, respectively. However, Subpopulation 2 coefficients of all individuals were between 0.963 and 1. The genotypes Coloradita and Rosada known as *S*. *tuberosum* Gp. Andigena genotypes and Oka 5880.22, known as *S*. *tarijense* (2x) genotype, were placed in subpopulation 2 which is a *S*. *tuberosum* Gp. Tuberosum (S2 Table).

**Fig 3:**
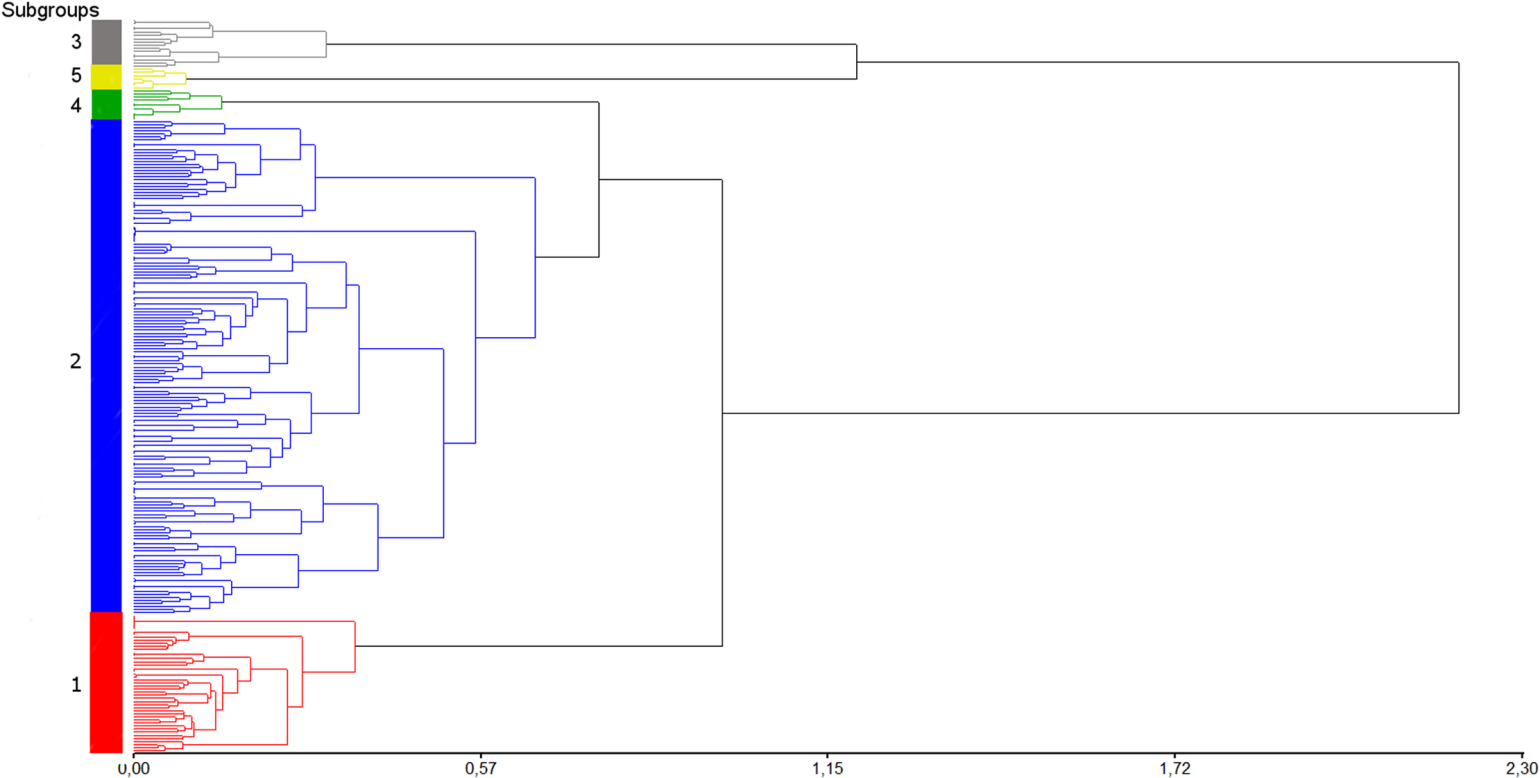
Dendrogram from Nei genetic distance matrix for 231 genotypes of the germplasm collection of INTA Balcarce potato breeding program. Dendrogram of the whole population divided in clusters. In the X axe are represented the genetic distances between groups and individuals. In the Y axe are represented the subpopulations in which the population was divided.

The dendrogram using Nei genetic distance among individuals of the whole population also revealed the presence of five clusters in the population. The assignment of the genotypes to the groups performed by the dendrogram, corresponded in 86% with the allocation made by the DAPC analysis (Fig 3). The same analysis performed within the most numerous subpopulations (1 and 2) divided the first one into 3 groups as did the DAPC analysis, with more than 70% coincidence in the assignment of genotypes to groups (Figure A in S2 Fig). Subpopulation 2 was divided into 4 groups, three of them matched more than 75% with DAPC analysis in the assignment of genotypes to groups. (Figure B in S2 Fig).

SNPZIP analysis detected 26 SNPs with the largest contribution to cluster identification. Two of them corresponded to LD 1, and the remaining 24 to the LD 2. Most of them are related to regions of the genome with known functions (S4 Table). The analysis was repeated for the largest *S*. *tuberosum* Gp. Tuberosum subpopulations (1 and 2) and identified 23 and 18 SNPs, respectively (S5 Table).

Results in Table 1 show that the genetic variability within populations (88%) was greater than the variability between populations (12%), which means that the population is genetically diverse. Allelic frequencies of subpopulations detected by DAPC were well differentiated (F_st_ = 0.118), and homozygotes and heterozygotes were balanced, (F_is_ = -0.022). The percentage of polymorphic loci was high across all populations. The genetic diversity of the whole population was high (H_o_ = 0.468, H_e_ = 0.390), but was higher in tetraploid subpopulations (1,2,3 and 4) than in the diploid subpopulation (5) (Table 2). Observed heterozygosity values (H_o_) were higher than expected (H_e_) for all the tetraploid subpopulations (1,2,3 and 4). They were similar in *S*. *tuberosum* Gp. Tuberosum subpopulations (H_o_ = 0.633, 0.614 and 0.594) but was a slightly lower in *S*. *tuberosum* Gp. Andigena subpopulation (H_o_ = 0.439). On the other hand, Subpopulation 5 (diploid) values were the lowest (H_o_ = 0.063). The groups detected within subpopulations 1 and 2 (three groups in both cases) were not well differentiated, which is supported by the low F_st_ values (0.01 and 0.008, respectively). Both subpopulations had F_is_ values close to zero, which means that there was no excess of homozygotes or heterozygotes (-0.093 y -0.033, respectively).

**Table 1:** Results of analysis of molecular variance (AMOVA) and F-statistics for the whole population and the most numerous Gp. Tuberosum subpopulations.

Population	SV	Df	SS	MS	Est.Var	%	F-statistics	p value
**Total**	**Among Pops**	4	53045.866	13261.467	167.582	12%	**F**_st_: 0.118	0.001
	**Within Pops**	473	590469.447	1248.350	1248.350	88%	**F**_is_: -0.022	0.954
	**Total**	477	643515.314		1415.932	100%		
**Subpopulation 1**	**Among Pops**	2	3180.36795	1590.183	12.236	1%	**F**_st_: 0.010	0.214
	**Within Pops**	127	159792.017	1258.204	1258.204	99%	**F**_is_: -0.093	0.997
	**Total**	129	162972.385		1270.441	100%		
**Subpopulation 2**	**Among Pops**	2	4213.340	2106.670	10.003	1%	**F**_st_: 0.008	0.017
	**Within Pops**	257	332369.245	1293.265	1293.266	99%	**F**_is_: -0.033	0.960
	**Total**	259	336582.585		1303.269	100%		

SV = Source of variation; Df = degrees of freedom; SS = Sum of squares; MS = Mean square; Est.Var. = Estimated variance; % = Percentage of variation.

**Table 2:** Statistics of genetic variation for the whole potato population.

Subpop.	N	H_o_		H_e_		%P
		Mean	SE	Mean	SE	
**1**	65	0.633	0.004	0.458	0.003	97.76%
**2**	134	0.614	0.003	0.469	0.002	99.81%
**3**	16	0.439	0.005	0.339	0.003	82.14%
**4**	17	0.594	0.005	0.411	0.003	90.62%
**5**	7	0.063	0.003	0.273	0.002	91.71%

Subpop. = Subpopulation, N = Number of individuals, H_o_ = Observed heterozygosity, H_e_ = Expected heterozygosity, %P = Percentage of polymorphic loci

The coefficient of genetic differentiation among populations (F_st_) (S6 Table) was high in tetraploid subpopulations (1,2,3 and 4) compared to the diploid subpopulation (5). In comparisons between tetraploid subpopulations formed by three groups of *S*. *tuberosum* Gp. Tuberosum (1, 2 and 4) and one group of *S*. *tuberosum* Gp. Andigena (3), the lowest value was found between subpopulations 2 and 3. F_st_ values between *S*. *tuberosum* Gp. Tuberosum subpopulations were low, suggesting low genetic differentiation among them.

### Pedigree information

Subpopulation 1 had 83% of INTA Balcarce breeding program clones and varieties. Serrana INTA had the highest value of MAC with 26.7% and appeared in the 17% of the individuals of the maternal path (S7 Table). MPI 59.703/21, B 2.63, Katahdin, Huinkul MAG and Saranac were the other clones with MAC values lower that Serrana INTA. Katahdin was the most frequent maternal ancestor of the subpopulation, it appeared in the 41% of the individuals, but its MAC was lower than that of Serrana INTA (11.66%). The most prominent paternal ancestor of the group was Huinkul MAG, with a MAC value of 31.75% and present in 39% of the individuals. Huinkul MAG is followed by Saranac, Earlaine and Katahdin. The latter is the most frequent paternal ancestor, but the MAC value is lower than that of Huinkul MAG.

Subpopulation 2 has 45% of individuals with wild species (*S*. *demissum*, *S*. *stoloniferum*, *S*. *verneii*, *S*. *phureja*) and *S*. *tuberosum Gp*. Andigena in their genetic background, which are INTA and CIP breeding clones and varieties from Argentina, United States, Netherlands and Chile. Also, subpopulation 2 has 34% of the CIP germplasm, originated from different breeding populations of recurrent selection which achieved resistance or tolerance to different stresses (B = Horizontal resistance to late blight, LTVR = Virus and heat tolerance, LBHT = Horizontal resistance to late blight and heat tolerance): 11% belongs to LTVR population, 42% belongs to B population and 0.06% belongs to LBHT population. The maternal and paternal ancestors that made the greatest contribution to the genotypes of the subpopulation are not so prominent. Conversely, there is a greater number of genotypes with small contributions. Katahdin is again the most frequent ancestor both in the maternal and paternal paths, but with very low values of MAC in both cases.

Subpopulation 3 is formed by *S*. *tuberosum* Gp. Andigena individuals and *S*. *tuberosum Gp*. Tuberosum CIP clones that belong to a CIP recurrent selection population called B1C5 with Gp. Andigena introgression and high level of horizontal resistance to late blight.

Subpopulation 4 maternal ancestors that made the greatest contribution were Gabriela, Katahdin, Saranac, USDA 96.56, Earlaine, CPC 1673–20 (adg) x Furore and Saskia. Their frequencies of appearance are very similar but the MAC values varied between 7.3 and 4%, Gabriela is the genotype with greater contribution. The paternal path is strongly influenced by Innovator who appeared in the 41% of the genotypes and its contribution to the group reaches the value of 50%. Katahdin is the most frequent genotype of the paternal path (60% of the individuals) but again its contribution to the genetic background of the group is very low (8.75%). Saskia x (CPC 1673–20 (adg) x Furore) and CPC 1673–11 (adg) x Furore are present also in the paternal background of this group in 53% of the individuals, with low contribution (7.03%).

The similarity matrix with the coefficients of relationship (CR) of each subpopulation presented low values of similarity between individuals of each group analyzed. The subpopulation A had only the 0.4% of the CR values above 0.5. Nevertheless, in the subpopulations B and D none of the CR values were greater than 0.5.

## Discussion

Genetic variability is essential to breeders for the generation of improved varieties resistant to diseases and adapted to adverse environmental conditions, without neglecting yield and industrial quality. Thus, it constitutes a source of new alleles for diverse complex traits of breeding interest contributing to broad potato genetic base. In the present study, population structure and genetic diversity was assessed in a diverse panel composed by genotypes that belong to two potato breeding programs with different strategies and final objectives, potato varieties from different countries around the world, wild species and *andigena* cultivars.

### Population structure and genetic diversity

DAPC analysis divided the population into well-defined clusters related to their genetic structure, which was associated with provenance, ploidy, taxonomy and breeding program of the genotypes. The groups generated are according to the different taxonomic backgrounds. This result was confirmed by a dendrogram of Nei distance of the whole population. The differences in the assignment of the genotypes to the groups between the two methods are based on the fact that dendrograms were constructed with Ward method, which is an agglomerative hierarchical method of clustering, and subpopulations were identified using DAPC which employs k-means with increasing number of clusters, a relocation non-hierarchical clustering method. Non-hierarchical methods begin partitioning samples into groups and iterate by moving them among groups to optimize measurements of within-group homogeneity and between-group heterogeneity. Those groups do not have hierarchical relationships between them [33]. The agglomerative hierarchical clustering begins with the assignation of each sample to its own cluster, then these are merged one at a time, and finally it ends up forming a large group with all the clusters [34]

Subpopulations composed by of *S*. *tuberosum* Gp. Tuberosum individuals had low differentiation among them. This result is in accordance with Hirsch et al., [14] who also found a strong structure in a diverse population formed by wild and cultivated potato species, but the substructure into cultivated potato was minimal, probably due to the similar North American germplasm background of the cultivated potato panel used in that experiment.

In addition, the subgroups into the most numerous *S*. *tuberosum* Gp. Tuberosum subpopulations were quite similar. According to this result, genetic background of subpopulations 1 and 2 could be quite homogeneous because they were generated from selection and crosses within breeding programs with a specific objective, consequently there could be no differences in allele frequencies within each subpopulation.

The SNPs that mostly contributed to the division of the population in clusters were those which mostly differ among the subpopulations. They were related to regions of the genome driving genetic divergence among groups [19]. Some of them are involved in regulation of transcription, carbohydrate metabolism and in different disease defense reaction chains [22, 23, 35, 36]. These results partially agree with [15] who found divergent allele frequencies for SNPs related to carbohydrate metabolism between diploid and tetraploid populations.

Heterozygosity values were higher in Group Tuberosum subpopulations than in the others. This may be related to the fact that the largest number of individuals in the total population are *S*. *tuberosum* Gp. Tuberosum. Also, the germplasm belonging to each of these subpopulations has a low coefficient of similarity between individuals. H_o_ value corresponded to Andigena subpopulation was lower than Tuberosum, probably because the Andigena subpopulation was formed by a small number of genotypes from the same place, which could represent a small sample of the total variability available from the Andigena Group. However, the lowest H_o_ value was that of diploid group, which could be a consequence of the small number of individuals that made up the subpopulation. This result is in agreement with Hirsch et al. [14] who suggest that greater ploidy may be associated with greater heterozygosity. On the other hand, the Illumina Infinium Potato SNP Array had developed using only elite potato germplasm [22], therefore polymorphisms belonging to Andigena and wild species could not be detected. Nevertheless, Berdugo-Cely et al., [37] found greater values of H_o_ in Andigena and Phureja populations using the Illumina Infinium Potato SNP Array but composed by a significant number of individuals.

### Pedigree information

The pedigree analysis into the *S*. *tuberosum* Gp. Tuberosum subpopulations detected the most frequent parental genotypes and those which did the most contribution to the subpopulations. In subpopulation 1, the maternal parent with the largest contribution was Serrana INTA which is an Argentinian variety known for its virus resistance and storage quality. It was widely used as progenitor in breeding programs for virus resistance at INTA Balcarce [38; 39] and CIP [40, 41]. On the paternal side, Huinkul MAG was the genotype with the largest contribution and high frequency of appearance. This is the first Argentinian cultivar (1948) and is known for its good storage and excellent culinary qualities. Until 1948, imported seed-tubers of varieties from Europe and North America were grown in Argentina. As of that year, Huinkul MAG displaced Katahdin, which occupied more than 80% of the area planted in the Southeast of the Province of Buenos Aires, the area with the highest potato production in the country [42]. According to the above, subpopulation 1 would be characterized by the history of the potato breeding program in Argentina. The pedigree analysis of subpopulation 2 detected many progenitors with low contributions and frequencies, in accordance to CIP´s breeding program’s strategy. The objective of CIP’s program is to develop advanced potato clones and varieties for the tropical, subtropical and temperate agro-ecologies of the developing world by means of the utilization of wide genetic resources [41]. Therefore, an attempt is made to look for genes of resistance and tolerance within the related *Solanum* species to introduce them into new potato varieties. In group 4, the main contributor and frequent paternal ancestor was Innovator. The genotype CPC 1673–20 (*adg*) was present in both paternal and maternal paths. This could be the reason why this subpopulation is the one that differs the most from the other two subpopulations of *S*. *tuberosum*, since there are not repeated progenitors with high frequency or contribution in the other two groups. Consequently, these might be the ones that were yielding the difference in the allelic frequencies with respect to the other groups of *S*. *tuberosum* Gp. Tuberosum. The genotype CPC 1673–20 was found among the first sources of resistance to *Globodera rostochiensis*, used in breeding programs [43, 44]. In addition, Innovator could be the ancestor who is conferring French fry processing characteristics to this subpopulation.

Nevertheless, Katahdin was present as the most frequent parental in both parental paths of all the *S*. *tuberosum* Gp. Tuberosum subpopulations analyzed with very low contributions to the genetic differentiation but contributing to the genetic background of all the groups. This variety was released in 1935 by the Beltsville USDA breeding program. It has been widely used in breeding programs in the United States and is found as a parent of most American cultivars of the 20^th^ century. Also, it is a recent ancestor and appears numerous times in the American cultivars pedigree [8, 45]. According to Love (1999) [45], this cultivar could be part of one fourth of the germplasm by which prominent North American cultivars are composed. The presence of Katahdin in the genetic background of all subpopulations analyzed, could be due to the fact that INTA Balcarce and CIP breeding programs began to develop after Katahdin introduction (1948 and 1974, respectively) and both of them used varieties from Europe and United States as part of the initial germplasm. [42,46].

DAPC and other statistical methods were used to assess plant population structure by different authors [37,47–48]. According to Rosyara et al., [48], STRUCTURE, EIGENSTRAT and DAPC are able to control population structure in association mapping studies, EIGENSTRAT and DAPC were slightly better than STRUCTURE but DAPC achieved better separation among groups. Pedigree analysis was used to complement the population assessment made from the SNPs. It provided data that allow a more complete characterization of the groups and helped to understand the structure of the population under study. The methodologies used in the present work and the SNPs molecular characterization, confirm that the population under study was structured into five clusters and this structure was clearly corresponded with the similarity in genetic background and breeding programs from where the varieties and breeding lines come. Alleles that were divergent among clusters are a guide to detect the principal differences due to breeding strategies and different origins among subpopulations. Information obtained in this work confirms that potato genetic base is broadening thanks to the particular efforts of many breeding programs and will allow breeders to design crossing strategies between parental groups aiming to maximize genetic diversity of a breeding program. Thus, this information aims to update knowledge about part of the existing potato germplasm and could be readily used as a guide for parental introduction in new breeding programs that want to combine contrasting variability sources such as the ones presented here.

## Supporting information

S1 Fig A) Graph of number of clusters vs. BIC. B): Graph of cross-validation of DAPC.
(PDF)

S2 Fig A) Dendrogram from Nei genetic distance matrix for subpopulation 1. B) Dendrogram from Nei genetic distance matrix for subpopulation 2.
(PDF)

S1 Table Genotypes that composed the potato panel. (PDF)

S2 Table Clusters formed by DAPC analysis. (PDF)

S3 Table Subgroups of genotypes resulting from the DAPC analysis for subpopulations 1 and 2. (PDF)

S4 Table Structural SNPs identified by SNPZIP analysis. (PDF)

S5 Table Structural SNPs identified by SNPZIP analysis, subpopulations groups 1 and 2. (PDF)

S6 Table Pairwise genetic differentiation values (Fst) between subpopulations of the whole potato panel. (PDF)

S7 Table Results of maximum average contribution calculated among the three *S*. *tuberosum* Gp. Tuberosum subpopulations. (PDF)
