# Supplementary material for: Discriminant analysis of principal components and pedigree assessment of genetic diversity and population structure in a tetraploid potato panel using SNPs
Source: PLoS One. 2018 Mar 16;13(3):e0194398. doi: 10.1371/journal.pone.0194398 (PMC5856401; doi:10.1371/journal.pone.0194398)
Supplement: S1 Fig — A) Graph of number of clusters vs. BIC. B): Graph of cross-validation of DAPC. (PDF) [file pone.0194398.s001.pdf]

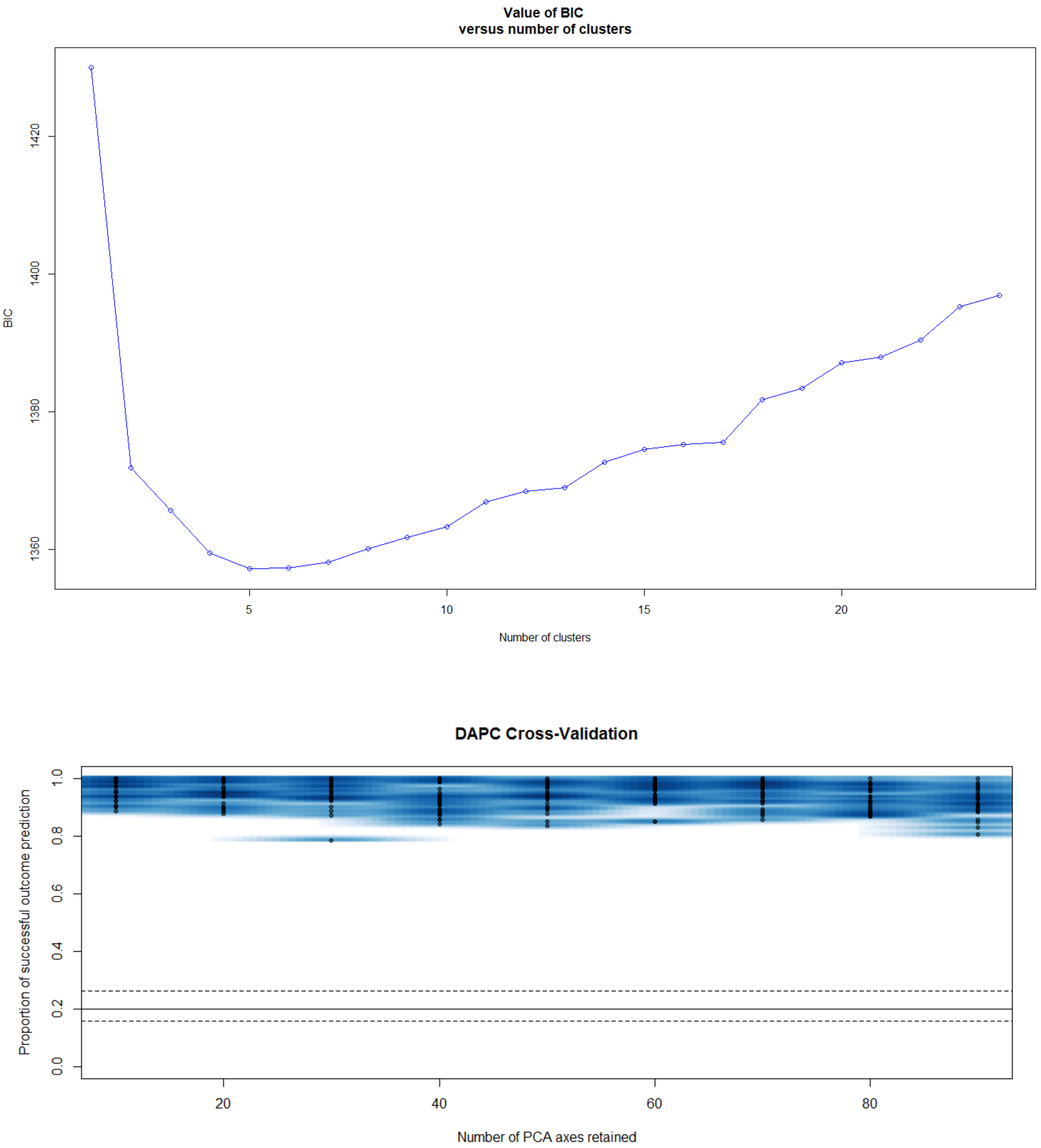

S3 Fig. (A) Graph of number of clusters vs. BIC. In the x axis is represented the different number of clusters that could be presented in the population. In the y axis, is represented the BIC value associated with each number of clusters. (B) Graph of cross-validation of DAPC. In the x axis is represented the number of PC retained in each DAPC. In the y axis, is represented the proportion of successful outcome prediction. Each dot represents the individual replicate of the analysis
