# Supplementary material for: Discriminant analysis of principal components and pedigree assessment of genetic diversity and population structure in a tetraploid potato panel using SNPs
Source: PLoS One. 2018 Mar 16;13(3):e0194398. doi: 10.1371/journal.pone.0194398 (PMC5856401; doi:10.1371/journal.pone.0194398)
Supplement: S2 Fig — A) Dendrogram from Nei genetic distance matrix for subpopulation 1. B) Dendrogram from Nei genetic distance matrix for subpopulation 2. (PDF) [file pone.0194398.s002.pdf]

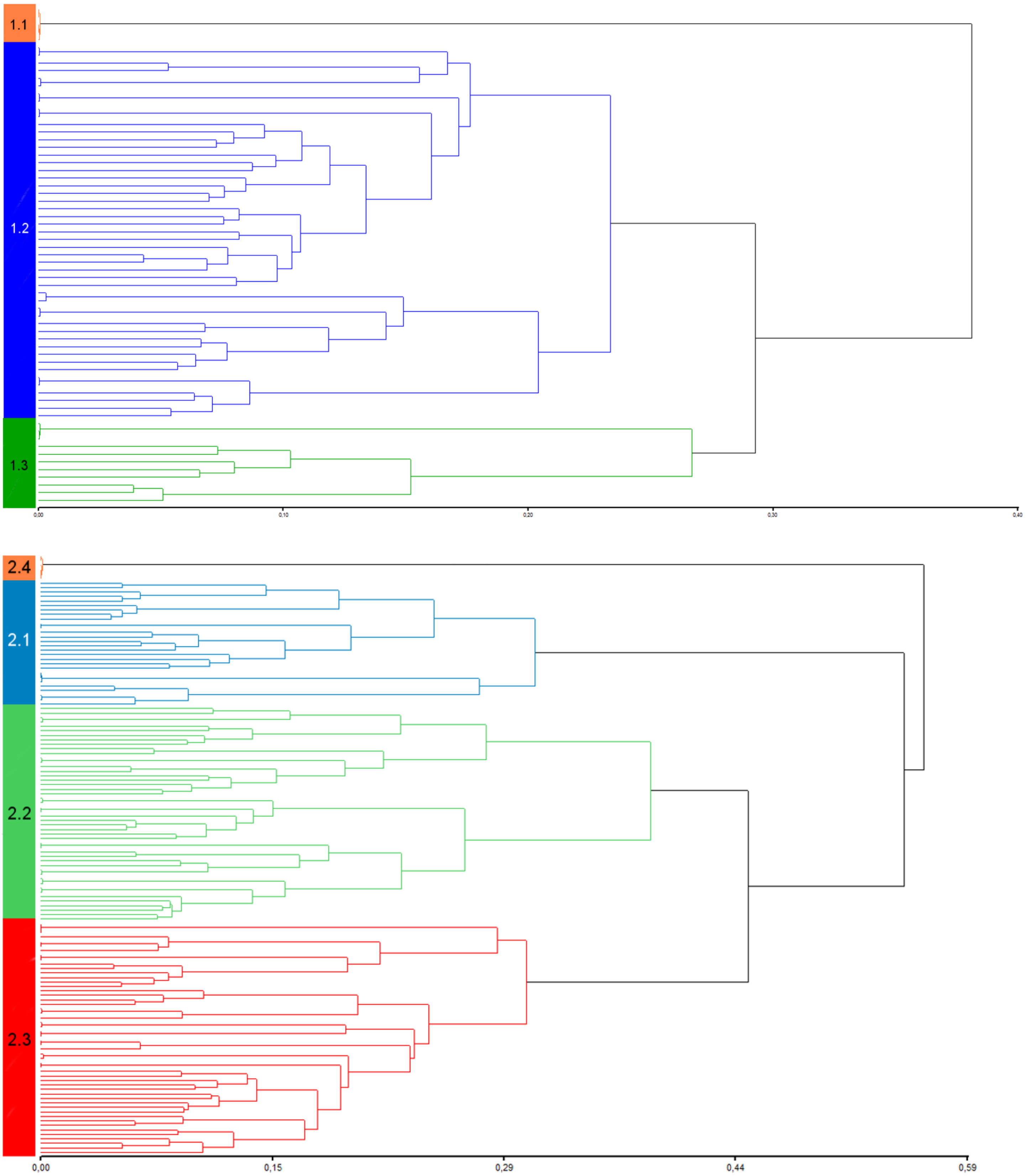

S5 Fig. A) Dendrogram from Nei genetic distance matrix for subpopulation 1. B) Dendrogram from Nei genetic distance matrix for subpopulation 2. In the X axe are represented the genetic distances between groups and individuals. In the Y axe are represented the subpopulations in which each population/subpopulation was divided
