# Supplementary material for: Discriminant analysis of principal components and pedigree assessment of genetic diversity and population structure in a tetraploid potato panel using SNPs
Source: PLoS One. 2018 Mar 16;13(3):e0194398. doi: 10.1371/journal.pone.0194398 (PMC5856401; doi:10.1371/journal.pone.0194398)
Supplement: S1 Table — (PDF) [file pone.0194398.s003.pdf]

S1 Table. Genotypes that composed the potato panel

Genotype	Origin	Species	Ploidy
21mat	Argentina	*Solanum Tuberosum* Gp. *Tuberosum x Solanum gourlayii*	4x
29mat	Argentina	*Solanum gourlayii x Solanum Tuberosum* Gp.*Tuberosum*	4x
304013.11	Peru	*Solanum Tuberosum* Gp. Tuberosum	4x
304013.18	Peru	*Solanum Tuberosum* Gp. Tuberosum	4x
304056.4	Peru	*Solanum Tuberosum* Gp. Tuberosum	4x
304072.6LB	Peru	*Solanum Tuberosum* Gp. Tuberosum	4x
304079.9	Peru	*Solanum Tuberosum* Gp. Tuberosum	4x
304081.2	Peru	*Solanum Tuberosum* Gp. Tuberosum	4x
304085.1	Peru	*Solanum Tuberosum* Gp. Tuberosum	4x
304092.1	Peru	*Solanum Tuberosum* Gp. Tuberosum	4x
304141.4	Peru	*Solanum Tuberosum* Gp. Tuberosum	4x
304146.1	Peru	*Solanum Tuberosum* Gp. Tuberosum	4x
304149.15	Peru	*Solanum Tuberosum* Gp. Tuberosum	4x
304150.11	Peru	*Solanum Tuberosum* Gp. Tuberosum	4x
304150.2	Peru	*Solanum Tuberosum* Gp. Tuberosum	4x
304150.8	Peru	*Solanum Tuberosum* Gp. Tuberosum	4x
304152.10	Peru	*Solanum Tuberosum* Gp. Tuberosum	4x
304152.5	Peru	*Solanum Tuberosum* Gp. Tuberosum	4x
304152.9LB	Peru	*Solanum Tuberosum* Gp. Tuberosum	4x
388615,22	Peru	*Solanum Tuberosum* Gp. Tuberosum	4x
388790.24	Peru	*Solanum Tuberosum* Gp. Tuberosum	4x
Tacna	Peru	*Solanum Tuberosum* Gp. Tuberosum	4x
391533.1	Peru	*Solanum Tuberosum* Gp. Tuberosum	4x
392785.31	Peru	*Solanum Tuberosum* Gp. Tuberosum	4x
392141.5	Peru	*Solanum Tuberosum* Gp. Tuberosum	4x
392785.24	Peru	*Solanum Tuberosum* Gp. Tuberosum	4x
393371.57	Peru	*Solanum Tuberosum* Gp. Tuberosum	4x
393371.66	Peru	*Solanum Tuberosum* Gp. Tuberosum	4x
393371.7	Peru	*Solanum Tuberosum* Gp. Tuberosum	4x
393072.22	Peru	*Solanum Tuberosum* Gp. Tuberosum	4x
393073.18	Peru	*Solanum Tuberosum* Gp. Tuberosum	4x
393073.26	Peru	*Solanum Tuberosum* Gp. Tuberosum	4x
393073.8	Peru	*Solanum Tuberosum* Gp. Tuberosum	4x
393075.15	Peru	*Solanum Tuberosum* Gp. Tuberosum	4x
393371.35	Peru	*Solanum Tuberosum* Gp. Tuberosum	4x
393371.37	Peru	*Solanum Tuberosum* Gp. Tuberosum	4x
393536.13	Peru	*Solanum Tuberosum* Gp. Tuberosum	4x
393595.1	Peru	*Solanum Tuberosum* Gp. Tuberosum	4x
395195.7	Peru	*Solanum Tuberosum* Gp. Tuberosum	4x
396004.225	Peru	*Solanum Tuberosum* Gp. Tuberosum	4x

Genotype	Origin	Species	Ploidy
396026.101	Peru	*Solanum Tuberosum* Gp. Tuberosum	4x
396031.108	Peru	*Solanum Tuberosum* Gp. Tuberosum	4x
396033.102	Peru	*Solanum Tuberosum* Gp. Tuberosum	4x
396037.215	Peru	*Solanum Tuberosum* Gp. Tuberosum	4x
397077.16	Peru	*Solanum Tuberosum* Gp. Tuberosum	4x
398017.54	Peru	*Solanum Tuberosum* Gp. Tuberosum	4x
398098.119	Peru	*Solanum Tuberosum* Gp. Tuberosum	4x
399001.16	Peru	*Solanum Tuberosum* Gp. Tuberosum	4x
399049.14	Peru	*Solanum Tuberosum* Gp. Tuberosum	4x
399053.15	Peru	*Solanum Tuberosum* Gp. Tuberosum	4x
399079.28	Peru	*Solanum Tuberosum* Gp. Tuberosum	4x
399083.4	Peru	*Solanum Tuberosum* Gp. Tuberosum	4x
86060	Peru	*Solanum Tuberosum* Gp. Tuberosum	4x
Granola	Peru	*Solanum Tuberosum* Gp. Tuberosum	4x
987174	Peru	*Solanum Tuberosum* Gp. Tuberosum	4x
Achirana	Argentina	*Solanum Tuberosum* Gp. Tuberosum	4x
Agata	Netherlands	*Solanum Tuberosum* Gp. Tuberosum	4x
Alpha	Netherlands	*Solanum Tuberosum* Gp. Tuberosum	4x
Ana	Brazil	*Solanum Tuberosum* Gp. Tuberosum	4x
Americana	Bolivia	*Solanum Tuberosum* Gp. Tuberosum	4x
Araucana	Argentina	*Solanum Tuberosum* Gp. Tuberosum	4x
Arazati	Uruguay	*Solanum Tuberosum* Gp. Tuberosum	4x
Astarte	Netherlands	*Solanum Tuberosum* Gp. Tuberosum	4x
Asterix	Netherlands	*Solanum Tuberosum* Gp. Tuberosum	4x
Atlantic	US.	*Solanum Tuberosum* Gp. Tuberosum	4x
Azul	Northwest of Argentina	*Solanum Tuberosum* Gp. Andigena	4x
B 00.607.1	Argentina	*Solanum Tuberosum* Gp. Tuberosum	4x
B 01.504.2	Argentina	*Solanum Tuberosum* Gp. Tuberosum	4x
B 01.505.2	Argentina	*Solanum Tuberosum* Gp. Tuberosum	4x
B 01.559.2	Argentina	*Solanum Tuberosum* Gp. Tuberosum	4x
B 02.556.2	Argentina	*Solanum Tuberosum* Gp. Tuberosum	4x
B 03.04.505.1	Argentina	*Solanum Tuberosum* Gp. Tuberosum	4x
B 03.04.525.1	Argentina	*Solanum Tuberosum* Gp. Tuberosum	4x
B 03.04.573.1	Argentina	*Solanum Tuberosum* Gp. Tuberosum	4x
B 03.04.573.3	Argentina	*Solanum Tuberosum* Gp. Tuberosum	4x
B 03.540.2	Argentina	*Solanum Tuberosum* Gp. Tuberosum	4x
B 03.559.1	Argentina	*Solanum Tuberosum* Gp. Tuberosum	4x
B 03.559.2	Argentina	*Solanum Tuberosum* Gp. Tuberosum	4x
B 03.565.7	Argentina	*Solanum Tuberosum* Gp. Tuberosum	4x
B 03.573.1	Argentina	*Solanum Tuberosum* Gp. Tuberosum	4x
B 03.574.1	Argentina	*Solanum Tuberosum* Gp. Tuberosum	4x
B 03.574.2	Argentina	*Solanum Tuberosum* Gp. Tuberosum	4x

Genotype	Origin	Species	Ploidy
B 03.578.1	Argentina	*Solanum Tuberosum* Gp. Tuberosum	4x
B 03.602.4	Argentina	*Solanum Tuberosum* Gp. Tuberosum	4x
B 03.620.1	Argentina	*Solanum Tuberosum* Gp. Tuberosum	4x
B 03.636.30 TT	Argentina	*Solanum Tuberosum* Gp. Tuberosum	4x
B 05.513.2	Argentina	*Solanum Tuberosum* Gp. Tuberosum	4x
B 06.07.640.1	Argentina	*Solanum Tuberosum* Gp. Tuberosum	4x
B 06.07.640.2	Argentina	*Solanum Tuberosum* Gp. Tuberosum	4x
B 06.07.804.2	Argentina	*Solanum Tuberosum* Gp. Tuberosum	4x
B 06.07.817.1	Argentina	*Solanum Tuberosum* Gp. Tuberosum	4x
B 06.559.1	Argentina	*Solanum Tuberosum* Gp. Tuberosum	4x
B 06.660.1	Argentina	*Solanum Tuberosum* Gp. Tuberosum	4x
B 06.07.640.6	Argentina	*Solanum Tuberosum* Gp. Tuberosum	4x
B 06.07.683.2	Argentina	*Solanum Tuberosum* Gp. Tuberosum	4x
B 06.665.1	Argentina	*Solanum Tuberosum* Gp. Tuberosum	4x
B 06.714.3	Argentina	*Solanum Tuberosum* Gp. Tuberosum	4x
B 06.785.2	Argentina	*Solanum Tuberosum* Gp. Tuberosum	4x
B 07.515.3	Argentina	*Solanum Tuberosum* Gp. Tuberosum	4x
B 07.516.1	Argentina	*Solanum Tuberosum* Gp. Tuberosum	4x
B 07.537.4	Argentina	*Solanum Tuberosum* Gp. Tuberosum	4x
B 07.573.1	Argentina	*Solanum Tuberosum* Gp. Tuberosum	4x
B 07.577.3	Argentina	*Solanum Tuberosum* Gp. Tuberosum	4x
B 07.591.2	Argentina	*Solanum Tuberosum* Gp. Tuberosum	4x
B 07.606.2	Argentina	*Solanum Tuberosum* Gp. Tuberosum	4x
B 07.606.4	Argentina	*Solanum Tuberosum* Gp. Tuberosum	4x
B 07.616.2	Argentina	*Solanum Tuberosum* Gp. Tuberosum	4x
B 07.660.1	Argentina	*Solanum Tuberosum* Gp. Tuberosum	4x
B 07.660.2	Argentina	*Solanum Tuberosum* Gp. Tuberosum	4x
B 78.502.5	Argentina	*Solanum Tuberosum* Gp. Tuberosum	4x
B 79.526.2	Argentina	*Solanum Tuberosum* Gp. Tuberosum	4x
B 79.571.1	Argentina	*Solanum Tuberosum* Gp. Tuberosum	4x
BT 84.527.48	Argentina	*Solanum Tuberosum* Gp. Tuberosum	4x
B 85.523.11	Argentina	*Solanum Tuberosum* Gp. Tuberosum	4x
B 85.616.3	Argentina	*Solanum Tuberosum* Gp. Tuberosum	4x
B 86.511.2LR	Argentina	*Solanum Tuberosum* Gp. Tuberosum	4x
B 86.525,1	Argentina	*Solanum Tuberosum* Gp. Tuberosum	4x
B 86.604.2LR	Argentina	*Solanum Tuberosum* Gp. Tuberosum	4x
B 87.605.2	Argentina	*Solanum Tuberosum* Gp. Tuberosum	4x
B 87.621.7	Argentina	*Solanum Tuberosum* Gp. Tuberosum	4x
B 87.823.1	Argentina	*Solanum Tuberosum* Gp. Tuberosum	4x
B 88.959.4	Argentina	*Solanum Tuberosum* Gp. Tuberosum	4x
B 90.519.2	Argentina	*Solanum Tuberosum* Gp. Tuberosum	4x
B 90.557.2	Argentina	*Solanum Tuberosum* Gp. Tuberosum	4x

Genotype	Origin	Species	Ploidy
B 90.557.2	Argentina	*Solanum Tuberosum* Gp. Tuberosum	4x
B 90.592.1	Argentina	*Solanum Tuberosum* Gp. Tuberosum	4x
B 90.610.4	Argentina	*Solanum Tuberosum* Gp. Tuberosum	4x
B 90.619.3	Argentina	*Solanum Tuberosum* Gp. Tuberosum	4x
B 90.827.1	Argentina	*Solanum Tuberosum* Gp. Tuberosum	4x
B 91.1042.2	Argentina	*Solanum Tuberosum* Gp. Tuberosum	4x
B_91.717.4	Argentina	*Solanum Tuberosum* Gp. Tuberosum	4x
B_91.880.3	Argentina	*Solanum Tuberosum* Gp. Tuberosum	4x
B 91.899.6	Argentina	*Solanum Tuberosum* Gp. Tuberosum	4x
B 92.10.1	Argentina	*Solanum Tuberosum* Gp. Tuberosum	4x
B 92.647.2	Argentina	*Solanum Tuberosum* Gp. Tuberosum	4x
B 92.659.2	Argentina	*Solanum Tuberosum* Gp. Tuberosum	4x
B 92.660.5	Argentina	*Solanum Tuberosum* Gp. Tuberosum	4x
B 92.678.4	Argentina	*Solanum Tuberosum* Gp. Tuberosum	4x
B 92.868.1	Argentina	*Solanum Tuberosum* Gp. Tuberosum	4x
B 92.903.4	Argentina	*Solanum Tuberosum* Gp. Tuberosum	4x
B 93.1104.4LR	Argentina	*Solanum Tuberosum* Gp. Tuberosum	4x
B 93.1116.3	Argentina	*Solanum Tuberosum* Gp. Tuberosum	4x
B 94.96.510.5	Argentina	*Solanum Tuberosum* Gp. Tuberosum	4x
B 97.523.4	Argentina	*Solanum Tuberosum* Gp. Tuberosum	4x
B 97.617.4	Argentina	*Solanum Tuberosum* Gp. Tuberosum	4x
B 98.99.508.1	Argentina	*Solanum Tuberosum* Gp. Tuberosum	4x
B 98.99.627.2	Argentina	*Solanum Tuberosum* Gp. Tuberosum	4x
B 99.558.1	Argentina	*Solanum Tuberosum* Gp. Tuberosum	4x
Balinca	Northwest of Argentina	*Solanum Tuberosum* Gp. Andigena	4x
Bannock russet	US.	*Solanum Tuberosum* Gp. Tuberosum	4x
Baronesa	Netherlands	*Solanum Tuberosum* Gp. Tuberosum	4x
Beate	Norway	*Solanum Tuberosum* Gp. Tuberosum	4x
BGRC-41479/1 (P1)	Argentina	*Solanum chacoense*	2x
BGRC-41479/15_(P15)	Argentina	*Solanum chacoense*	2x
Bintje	Netherlands	*Solanum Tuberosum* Gp. Tuberosum	4x
Blanca dulce	Northwest of Argentina	*Solanum Tuberosum* Gp. Andigena	4x
Bonaerense La Ballenera	Argentina	*Solanum Tuberosum* Gp. Tuberosum	
BT 84.529.5	Argentina	*Solanum Tuberosum* Gp. Tuberosum	4x
BT 84.530.28	Argentina	*Solanum Tuberosum* Gp. Tuberosum	4x
BT 85.520.117	Argentina	*Solanum Tuberosum* Gp. Tuberosum	4x
Calén INTA	Argentina	*Solanum Tuberosum* Gp. Tuberosum	4x
Chacay INTA	Argentina	*Solanum Tuberosum* Gp. Tuberosum	4x
Chieftain	US.	*Solanum Tuberosum* Gp. Tuberosum	4x
Collareja	Northwest of Argentina	*Solanum Tuberosum* Gp. Andigena	4x
Collareja de Jujuy	Northwest of Argentina	*Solanum Tuberosum* Gp. Andigena	4x

Genotype	Origin	Species	Ploidy
Coloradita	Northwest of Argentina	*Solanum Tuberosum* Gp. Andigena	4x
Cuarentona	Northwest of Argentina	*Solanum Tuberosum* Gp. Andigena	4x
Daekwar.48	Korea	*Solanum Tuberosum* Gp. Tuberosum	4x
E_86.011	Peru	*Solanum Tuberosum* Gp. Tuberosum	4x
Eldorado	UK	*Solanum Tuberosum* Gp. Tuberosum	4x
Eurostar	Netherlands	*Solanum Tuberosum* Gp. Tuberosum	4x
Feiwu	China	*Solanum Tuberosum* Gp. Tuberosum	4x
Fenchuixue	China	*Solanum Tuberosum* Gp. Tuberosum	4x
FL 1879	US.	*Solanum Tuberosum* Gp. Tuberosum	4x
Fontane	Netherlands	*Solanum Tuberosum* Gp. Tuberosum	4x
Frital INTA	Argentina	*Solanum Tuberosum* Gp. Tuberosum	4x
Gem Russet	US.	*Solanum Tuberosum* Gp. Tuberosum	4x
Huinkul	Argentina	*Solanum Tuberosum* Gp. Tuberosum	4x
Innovator	Netherlands	*Solanum Tuberosum* Gp. Tuberosum	4x
Iporá	Uruguay	*Solanum Tuberosum* Gp. Tuberosum	4x
Jopung	Korea	*Solanum Tuberosum* Gp. Tuberosum	4x
Kantara	Netherlands	*Solanum Tuberosum* Gp. Tuberosum	4x
Kardal	Netherlands	*Solanum Tuberosum* Gp. Tuberosum	4x
Karu	Chile	*Solanum Tuberosum* Gp. Tuberosum	4x
Keluné	Argentina	*Solanum Tuberosum* Gp. Tuberosum	4x
Kennebec	US.	*Solanum Tuberosum* Gp. Tuberosum	4x
Kexin	China	*Solanum Tuberosum* Gp. Tuberosum	4x
La Florida	Argentina	*Solanum Tuberosum* Gp. Tuberosum	4x
M. roja	-	*Solanum Tuberosum* Gp. Tuberosum	4x
Monalisa	Netherlands	*Solanum Tuberosum* Gp. Tuberosum	4x
Morada Morada	Northwest of Argentina	*Solanum Tuberosum* Gp. Tuberosum	4x
Moradita	Northwest of Argentina	*Solanum Tuberosum* Gp. Tuberosum	4x
Newen INTA	Argentina	*Solanum Tuberosum* Gp. Tuberosum	4x
Nicola	Germany	*Solanum Tuberosum* Gp. Tuberosum	4x
OCL 7383.7	Northwest of Argentina	*Solanum tarijense*	2x
OCL 7383.12	Northwest of Argentina	*Solanum tarijense*	
Oka 5632.11	Northwest of Argentina	*Solanum tarijense*	
Oka 5880.22	Northwest of Argentina	*Solanum tarijense*	
Ona INIA	Chile	*Solanum Tuberosum* Gp. Tuberosum	4x
Overa 213	Northwest of Argentina	*Solanum Tuberosum* Gp. Andigena	4x
Pampeana INTA	Argentina	*Solanum Tuberosum* Gp. Tuberosum	4x
Pehuenche	Chile	*Solanum Tuberosum* Gp. Tuberosum	4x
Pentland Crown	UK	*Solanum Tuberosum* Gp. Tuberosum	4x
Pintada	Northwest of Argentina	*Solanum Tuberosum* Gp. Andigena	4x

Genotype	Origin	Species	Ploidy
PO 97.11.10	Argentina	*Solanum Tuberosum* Gp. Tuberosum	4x
PO 97.11.9	Argentina	*Solanum Tuberosum* Gp. Tuberosum	4x
PO 99.26.1	Argentina	*Solanum Tuberosum* Gp. Tuberosum	4x
Poluya	Bolivia	*Solanum stenotomum*	2x
Primicia	Argentina	*Solanum Tuberosum* Gp. Tuberosum	4x
Pukará	Chile	*Solanum Tuberosum* Gp. Tuberosum	4x
Puren	Chile	*Solanum Tuberosum* Gp. Tuberosum	4x
Purple Majesty	US.	*Solanum Tuberosum* Gp. Tuberosum	4x
Ramos	Netherlands	*Solanum Tuberosum* Gp. Tuberosum	4x
Ranger Russet	US.	*Solanum Tuberosum* Gp. Tuberosum	4x
Revolución	Perú	*Solanum Tuberosum* Gp. Tuberosum	4x
Rosada	Northwest of Argentina	*Solanum Tuberosum* Gp. Andigena	4x
Russet Burbank	US	*Solanum Tuberosum* Gp. Tuberosum	4x
RZ 90.44.3	Netherlands	*Solanum Tuberosum* Gp. Tuberosum	4x
Sani	Northwest of Argentina	*Solanum Tuberosum* Gp. Andigena	4x
Shepody	Canada	*Solanum Tuberosum* Gp. Tuberosum	4x
Sierra volcán	Argentina	*Solanum Tuberosum* Gp. Tuberosum	4x
Snowden	US.	*Solanum Tuberosum* Gp. Tuberosum	4x
Spunta	Netherlands	*Solanum Tuberosum* Gp. Tuberosum	4x
Tuni blanca 105	Northwest of Argentina	*Solanum Tuberosum* Gp. Andigena	4x
Umatilla	US.	*Solanum Tuberosum* Gp. Tuberosum	4x
Unknown	.	*Solanum Tuberosum* Gp. Tuberosum	4x
Yaguarí	Uruguay	*Solanum Tuberosum* Gp. Tuberosum	4x
Yagana	Chile	*Solanum Tuberosum* Gp. Tuberosum	4x
