# Supplementary material for: Discriminant analysis of principal components and pedigree assessment of genetic diversity and population structure in a tetraploid potato panel using SNPs
Source: PLoS One. 2018 Mar 16;13(3):e0194398. doi: 10.1371/journal.pone.0194398 (PMC5856401; doi:10.1371/journal.pone.0194398)
Supplement: S2 Table — (PDF) [file pone.0194398.s004.pdf]

S2 Table. Clusters formed by DAPC analysis

Subpopulation 1			Subpopulation 2					Subpop. 3	Subpop. 4	Subpop. 5
21 mat	B 88.959.4	B 90.610.4	304056.4	396037.215	B 92.10.1	Poluya	M. roja	399001.16	396004.225	393075.15
29 mat	B 90.519.2	Bonaerense La Ballenera	304072.6LB	397077.16	B 92.660.5	Pukará	Oka 5880.22	399049.14	B 00.607.1	BGRC-41479/1 (P1)
304013.11	B 90.557.2	BT 84.527.48	304079.9	398017.54	B 92.868.1	Puren	Purple Majesty	399053.15	B 01.504.2	OCL 7383.7
304013.18	B 90.557.2	BT 84.530.28	304081.2	398098119	B 93.1104.4LR	Ramos	Revolución	Balinca	B 03.04.573.1	Pera o Señorita
304085.1	B 90.619.3	Sierra Volcán	304141.4	399079.28	B 93.1116.3	Ranger Russet	Rosada	Blanca dulce	B 03.559.1	BGRC-41479/15 (P15)
304092.1	B 90.827.1		304146.1	399083.4	B 94.96.510.5	RZ 90.44.3	Yaguarí	Collareja	B 03.573.1	OCL 7383.122
392785.31	B 91.717.4		304149.15	800959 (Granola)	B 97.523.4	Snowden		Collareja de Jujuy	B 03.574.1	Oka 5632.112
393371.35	B 91.880.3		304150.11	987174	B 97.617.4	Spunta		Cuarentona	B 03.574.2	
Araucana	B 92.647.2		304150.2	Achirana	B 99.558.1	Umatilla		Morada Morada	B 07.577.3	
B 01.559.2	B 92.659.2		304150.8	Agata	Bannock russet	Unknown		Moradita	Innovator	
B 02.556.2	B 92.903.4		304152.10	Alpha	BT 84.529.5	Yagana		Overa	PO 97.11.10	
B 03.540.2	B 98.99.508.1		304152.5	Ana	Chieftain	393595.1		Sani	PO 97.11.9	
B 03.578.1	B 98.99.627.2		304152.9 LB	Astarte	Feiwu	395195.7		Tuni blanca 105	B 03.04.573.3	
B 03.636.30TT	BT 85.520.117		388615.22	Asterix	Fenchuixue	396033102		Azul	B 07.606.2	
B 05.513.2	Calen INTA		388790.24	B 03.04.505.1	FL 1879	86060		Moradita	Eurostar	
B 06.07.640.1	Chacay Inta		390478.9 (Tacna)	B 03.559.2	Frital	Americana		Pintada	PO 99.26.1	
B 06.07.640.2	Daekwar.48		391533.1	B 03.565.7	Gem Russet	Arazati			Shepody	
B 06.07.804.2	E 86.011		392141.5	B 07.515.3	Iporá	Atlantic				
B 06.07.817.1	Fontane		392785.24	B 07.516.1	Kantara	B 03.04.525.1				
B 06.660.1	Huinkul		393371.57	B 07.616.2	Kardal	B 03.620.1				
B 06.665.1	Kennebec		393371.66	B 07.660.1	Karu	B 79.526.2				
B 06.714.3	Pampeana		393371.7	B 07.660.2	Kelun	B 85.523.11				
B 06.785.2	Primicia		393072.22	B 79.571.1	Kexin	B 92.678.4				
B 07.537.4	Russet Burbank		393073.18	B 85.616.3	La Florida	Baronesa				
B 07.591.2	B 01.505.2		393073.26	B 86.525,1	Monalisa	Beate				
B 07.606.4	B 03.602.4		393073.8	B 86.604.2LR	Newen	Bintje				
B 78.502.5	B 06.07.640.6		393371.37	B 87.605.2	Nicola	BT 84.529.5				
B 86.511.2LR	B 06.07.683.2		393536.13	B 90.592.1	Ona	Coloradita				
B 87.621.7	B 06.559.1		396026.101	B 91.1042.2	Pehuenche	Eldorado				
B 87.823.1	B 07.573.1		396031.108	B 91.899.6	Pentland Crown	Jopung				
