# Supplementary material for: Discriminant analysis of principal components and pedigree assessment of genetic diversity and population structure in a tetraploid potato panel using SNPs
Source: PLoS One. 2018 Mar 16;13(3):e0194398. doi: 10.1371/journal.pone.0194398 (PMC5856401; doi:10.1371/journal.pone.0194398)
Supplement: S3 Table — (PDF) [file pone.0194398.s005.pdf]

S4 Table. Subgroups of genotypes resulting from the DAPC analysis for subpopulations 1 and 2.

Subpopulation 1			Subpopulation 2		
Subgroup 1.1	*Subgroup 1.2*	*Subgroup 1.3*	*Subgroup 2.1*	*Subgroup 2.2*	*Subgroup 2.3*
304013.11	304013.18	21 mat	304056.4	304141.4	304072.6LB
B 01.559.2	304092.1	29 mat	304079.9	304146.1	304150.11
B 03.636.30 TT	392785.31	304085.1	304081.2	304152.5	304150.8
B 05.513.2	393371.35	Araucana	304149.15	388615.22	304152.10
B 06.07. 640.1	Pampeana INTA	B 02.556.2	304150.2	388790.24	393073.18
B 06.07.640.2		B 03.540.2	304152.9 LB	390478.9 (Tacna)	399079.28
B 06.07.817.1		B 03.578.1	391533.1	392141.5	800959 (Granola)
B 06.660.1		B 06.07.804.2	392785.24	399083.4	Achirana
B 06.665.1		B 06.714.3	393371.57	987174	Agata
B 07.537.4		B 06.785.2	393371.66	B 03.559.2	Alpha
B 07.591.2		B 87.621.7	393371.7	B 07.515.3	Ana
B 07.606.4		B 87.823.1	393072.22	B 07.516.1	Asterix
B 78.502.5		B 88.959.4	393073.26	B 07.616.2	B 03.04.505.1
B 86.511.2LR		B 90.519.2	393073.8	B 07.660.1	B 03.565.7
B 90.557.2		B 90.619.3	393371.37	B 07.660.2	B 79.571.1
B 90.557.2		B 91.717.4	393536.13	B 86.525,1	B 85.616.3
B 90.827.1		B 91.880.3	396026.101	B 90.592.1	B 86.604.2LR
B 92.647.2		B 92.659.2	396031.108	B 91.1042.2	B 87.605.2
B 98.99.508.1		B 92.903.4	396037.215	B 91.899.6	B 92.10.1
B 98.99.627.2		Calén INTA	397077.16	B 92.868.1	B 92.660.5
BT 85.520.117		Chacay INTA	398017.54	B 93.1116.3	Feiwu
Huinkul		Daekwar.48	398098.119	B 97.523.4	Fenchuixue
Kennebec		E 86.011	Astarte	B 97.617.4	Kardal
Primicia		Fontane	B 93.1104.4LR	B 99.558.1	Karu
B 01.505.2		Russet Burbank	B 94.96.510.5	Bannock russet	Keluné
B 06.07.640.6		B 03.602.4	BT 84.529.5	Chieftain	La Florida
B 06.07.683.2		B 07.573.1	Iporá	FL 1879	Monalisa
B 06.559.1		B 90.610.4	Spunta	Frital	Nicola
Bonaerense La Ballenera		BT 84.527.48	Unknown	Gem Russet	Ona INIA
Sierra Volcán		BT 84.530.28	396033.102	Kantara	Pehuenche
			86060	Kexin	Pentland Crown
			Oka 5880.22	Newen	Poluya
			Revolución	Puren	Pukará
				Ranger Russet	Ramos
				RZ 90.44.3	Yagana
				Snowden	B 79.526.2
				Umatilla	B 85.523.11
				393595.1	B 92.678.4
				395195.7	Baronesa
				Americana	Beate
				Arazati	Bintje
				Atlantic	Coloradita
				B 03.04.525.1	Eldorado
				B 03.620.1	Rosada
				Jopung	
				M. roja	
				Purple Majesty	
				Yaguarí	
