# Supplementary material for: Discriminant analysis of principal components and pedigree assessment of genetic diversity and population structure in a tetraploid potato panel using SNPs
Source: PLoS One. 2018 Mar 16;13(3):e0194398. doi: 10.1371/journal.pone.0194398 (PMC5856401; doi:10.1371/journal.pone.0194398)
Supplement: S4 Table — (PDF) [file pone.0194398.s006.pdf]

S6 Table. Structural SNPs identified by SNPZIP analysis. Annotations were obtained from the Potato Genome database and associated functions were obtained from Gene Ontology (GO) database

SNP id	Chr	LD	Contribution	Annotation	Gene ontology annotations
solcap_snp_c2_16299	12	1	0.004371293	Acyl-CoA thioesterase	Hydrolysis of acyl-CoAs
solcap_snp_c2_31600	6		0.006005380	Conserved gene of unknown function	
solcap_snp_c1_14248	1	2	0.002476263	Calmodulin-binding transcription activator (Camta), plants	Transcription factor
solcap_snp_c1_22	1		0.003083547	Gene of unknown function	
solcap_snp_c1_3436	4		0.003365768	Ubiquinone biosynthesis protein coq-8	Ubiquinone biosynthetic process
solcap_snp_c1_3497	4		0.002568070	Gene of unknown function	
solcap_snp_c1_7227	10		0.003163455	Gene of unknown function	
solcap_snp_c2_23337	12		0.003222444	Ethylene-responsive element binding protein	Regulation of transcription, DNA-templated
solcap_snp_c2_27765	9		0.004587061	Sucrose synthase	Carbohydrate metabolism
solcap_snp_c2_28761	10		0.002698637	Gene of unknown function	
solcap_snp_c2_30950	2		0.002465777	Zinc finger protein	Transcription factor
solcap_snp_c2_31358	4		0.004142081	Calcium dependent protein kinase	Protein phosphorylation, defense response
solcap_snp_c2_31360	4		0.003550113	Calcium dependent protein kinase	Protein phosphorylation, defense response
solcap_snp_c2_31600	6		0.004857531	Conserved gene of unknown function	
solcap_snp_c2_34197	11		0.002453164	Protein kinase	Protein phosphorylation
solcap_snp_c2_38246	4		0.002452655	Gene of unknown function	
solcap_snp_c2_41906	2		0.002608736	Zinc/iron transporter	Metal ion transmembrane transporter
solcap_snp_c2_46707	7		0.002866617	4-cumarate-COA-ligase	Catalytic activity, defense response
solcap_snp_c2_46710	7		0.002909402	AMP dependent CoA ligase	Catalytic activity, defense response
solcap_snp_c2_46715	7		0.002948732	Porin/voltage-dependent anion-selective channel protein	ATPase activity, coupled to transmembrane movement of substances
solcap_snp_c2_47510	3		0.002903629	Component of high affinity nitrate transporter	Nitrate transport
solcap_snp_c2_48715	7		0.002840265	Purine permease 3	Transporter activity
solcap_snp_c2_49068	2		0.002911321	Metalloprotease m41 ftsh	Microtubule-severing ATPase activity
solcap_snp_c2_51559	4		0.002673901	Lycopene beta cyclase, chloroplastic	Carotenoid biosynthetic process
solcap_snp_c2_52663	7		0.002630232	Acetyltransferase	N-acetyltransferase activity
solcap_snp_c2_54069	0		0.003111650	Sn-1 protein	Defense response
