# Supplementary material for: Discriminant analysis of principal components and pedigree assessment of genetic diversity and population structure in a tetraploid potato panel using SNPs
Source: PLoS One. 2018 Mar 16;13(3):e0194398. doi: 10.1371/journal.pone.0194398 (PMC5856401; doi:10.1371/journal.pone.0194398)
Supplement: S5 Table — (PDF) [file pone.0194398.s007.pdf]

S7 Table. Structural SNPs identified by SNPZIP analysis, into Subpopulations 1 and 2. Annotations were obtained from the Potato Genome database and associated functions were obtained from Gene Ontology (GO) database

Pop	SNP id	Chr	DA	Contribution	Annotation	Gene ontology annotations
1	solcap_snp_c1_11750	4	1	0.002323946	Fructose-1,6-bisphosphatase, cytosolic	Carbohydrate metabolic process
	solcap_snp_c1_12899	11		0.002512040	PHAP2A protein	Transcription factor activity, sequence-specific DNA binding
	solcap_snp_c1_13530	10		0.002877826	Calcium-dependent protein kinase 4	Protein phosphorylation, defense reactions
	solcap_snp_c1_13531	10		0.003255512	Calcium-dependent protein kinase 4	Protein phosphorylation, defense reactions
	solcap_snp_c2_11313	6		0.002266546	ATP-dependent helicase	ATP-dependent RNA helicase
	solcap_snp_c2_12106	1		0.003089240	SPX (SYG1/Pho81/XPR1) domain-containing protein	ATPase activity, coupled to transmembrane movement of substances
	solcap_snp_c2_12252	11		0.002582589	Tetratricopeptide repeat (TPR)-containing protein	Regulation of different cellular functions
	solcap_snp_c2_15331	11		0.002983387	Auxin response factor	Regulation of transcription, auxin-activated signaling pathway
	solcap_snp_c2_27571	6		0.002253724	Thiamine biosynthesis protein ThiC variant L1	Thiamine biosynthetic process
	solcap_snp_c2_27773	12		0.003012334	Conserved gene of unknown function	
	solcap_snp_c2_27831	10		0.002353000	Conserved gene of unknown function	
	solcap_snp_c2_32359	1		0.002401549	Conserved gene of unknown function	
	solcap_snp_c2_32954	11		0.002655819	Mitochondrial prohibitin 1	Mitochondrial inner membrane
	solcap_snp_c2_37119	3		0.002395287	Phytoene dehydrogenase, chloroplastic/chromoplastic	Carotenoid biosynthetic pathway
	solcap_snp_c2_39801	4		0.002368024	Fructose-1,6-bisphosphatase, cytosolic	Carbohydrate metabolic process
	solcap_snp_c2_4225	11		0.002827223	Conserved gene of unknown function	
	solcap_snp_c2_53946	10		0.002335623	Phenylalanine ammonia-lyase 1	Phenylpropanoid metabolism
	solcap_snp_c2_54932	10		0.002315496	Serine-threonine protein kinase, plant-type	Signaling and plant defense
	solcap_snp_c2_55279	3		0.003206189	Pectin methylesterase 1	Pectin methylation
	solcap_snp_c1_10941	4	2	0.003623051	F-box protein SKIP14	Protein ubiquitination
	solcap_snp_c1_12887	4		0.003529604	Conserved gene of unknown function	
	solcap_snp_c1_16261	4		0.004247470	Sentrin/sumo-specific protease	Protease
	solcap_snp_c2_51372	8		0.003273088	Gene of unknown function	
2	solcap_snp_c1_13641	6	1	0.002547742	WERBP-1 protein	Regulation of transcription, DNA-templated
	solcap_snp_c1_1535	0		0.004301551	Calcium-dependent protein kinase	Protein phosphorylation, defense response
	solcap_snp_c1_2065	6		0.002478365	Phosphate abc transporter	Transport
	solcap_snp_c1_7148	10		0.002732923	RBP50	mRNA processing
	solcap_snp_c1_7187	10		0.002582318	RING finger	Protein ubiquitination
	solcap_snp_c1_7192	10		0.003123266	Actin	Nucleotide binding
	solcap_snp_c1_7520	7		0.002433071	Rhamnose:beta-solanine/beta-chaconine rhamnosyltransferase	Transferase activity
	solcap_snp_c2_17592	1		0.002511684	Conserved gene of unknown function	
	solcap_snp_c2_24297	6		0.002499110	Conserved gene of unknown function	
	solcap_snp_c2_29745	10		0.002775460	Transferase, transferring glycosyl groups	Galactosyltransferase activity
	solcap_snp_c2_45770	8		0.002445928	Catalytic	Catalytic activity
	solcap_snp_c2_46398	7		0.002588965	Protein kinase APK1B, chloroplast	Protein phosphorylation
	solcap_snp_c2_54353	1		0.002977316	DNA polymerase eta	DNA repair
	solcap_snp_c2_55831	7		0.003099008	Sucrose-phosphate-synthase	Sucrose biosynthetic process
	solcap_snp_c2_5864	6		0.003113798	Protein CASP	Integral component of membrane
	solcap_snp_c2_10409	5	2	0.004274992	Ferritin	Ferroxidase activity, iron ion transport
	solcap_snp_c2_24595	12		0.007038564	Gene of unknown function	
	solcap_snp_c2_33893	6		0.003990978	Cytochrome c1-1, heme protein, mitochondrial	Electron carrier activity, respiratory chain
