# Supplementary material for: Discriminant analysis of principal components and pedigree assessment of genetic diversity and population structure in a tetraploid potato panel using SNPs
Source: PLoS One. 2018 Mar 16;13(3):e0194398. doi: 10.1371/journal.pone.0194398 (PMC5856401; doi:10.1371/journal.pone.0194398)
Supplement: S6 Table — (PDF) [file pone.0194398.s008.pdf]

S8 Table. Pairwise genetic differentiation values (Fst) between subpopulations of the whole potato panel.

**Subpop.**	**Subpop.**	**Fst**	**Nm**	**pvalue**	**permutations**
1	2	0.023	10.460	0.000	9999
1	3	0.110	2.016	0.000	9999
2	3	0.073	3.180	0.000	9999
1	4	0.078	2.975	0.000	9999
2	4	0.054	4.392	0.000	9999
3	4	0.135	1.595	0.000	9999
1	5	0.491	0.259	0.000	9999
2	5	0.475	0.277	0.000	9999
3	5	0.567	0.191	0.000	9999
4	5	0.536	0.217	0.000	9999
