# Supplementary material for: Discriminant analysis of principal components and pedigree assessment of genetic diversity and population structure in a tetraploid potato panel using SNPs
Source: PLoS One. 2018 Mar 16;13(3):e0194398. doi: 10.1371/journal.pone.0194398 (PMC5856401; doi:10.1371/journal.pone.0194398)
Supplement: S7 Table — (PDF) [file pone.0194398.s009.pdf]

S9 Table. Results of Maximum average contribution calculated among the three *S. tuberosum* Gp. Tuberosum subpopulations.

Number of ancestors: Ancestors that were considered in the genealogy of each individual of the subpopulation. Type: Maternal or paternal line. MCP: Maximum Cumulative probability: Sum of the maximum probabilities provided by the ancestor in relation to the level of the genealogy in which it occurs in each genotype of the subpopulation. FA: Frequency of appearance: of the ancestor in the individuals of the subpopulation. MAC: Maximum average contribution: MCP/FA

Group	Number of ancestors considered	Path	Ancestors	MCP	FA	FA (%)	MAC	MAC (%)
1 (N=64)	347	Maternal	Serrana INTA	2.9375	11	17.1875	0.267045455	26.70454545
			MPI 59.703/21	1.46875	11	17.1875	0.133522727	13.35227273
			B 2.63	1.78125	14	21.875	0.127232143	12.72321429
			Katahdin	3.03125	26	40.625	0.116586538	11.65865385
			Huinkul	2.84375	25	39.0625	0.11375	11.375
			Saranac	2.28125	22	34.375	0.103693182	10.36931818
	266	Paternal	Katahdin	5	38	59.375	0.131578947	13.15789474
			Earlaine	5.21875	31	48.4375	0.168346774	16.83467742
			Saranac	4.84375	27	42.1875	0.179398148	17.93981481
			Huinkul	7.9375	25	39.0625	0.3175	31.75
2 (N=128)	517	Maternal	Serrana INTA	4.25	10	7.8125	0.425	42.5
			387170.16	3	8	6.25	0.375	37.5
			MPI 59.703/21	2.15625	11	8.59375	0.196022727	19.60227273
			B 2.63	2.28125	13	10.15625	0.175480769	17.54807692
			383300,21 (C83.119)	2.125	13	10.15625	0.163461538	16.34615385
			575049 (CEW-69-1)	1.4375	9	7.03125	0.159722222	15.97222222
			386614,16 (XY16)	1.125	8	6.25	0.140625	14.0625
			Huinkul	2.40625	19	14.84375	0.126644737	12.66447368
			MPI 55.957/54	1.0625	10	7.8125	0.10625	10.625
			Katahdin	2.484375	42	32.8125	0.059151786	5.915178571
	518	Paternal	389746,2	4.25	9	7.03125	0.472222222	47.22222222
			Huinkul	3.0625	9	7.03125	0.340277778	34.02777778
			Desiree	3.6875	11	8.59375	0.335227273	33.52272727
			386614,16 (XY 16)	3.125	11	8.59375	0.284090909	28.40909091
			381379,9	2.125	9	7.03125	0.236111111	23.61111111
			Urgenta	1.8125	9	7.03125	0.201388889	20.13888889
			Depesche	1.8125	9	7.03125	0.201388889	20.13888889
			383300,21 (C83.119)	1.625	9	7.03125	0.180555556	18.05555556
			Y84.007	2.0625	13	10.15625	0.158653846	15.86538462
			Atlantic	2.0625	13	10.15625	0.158653846	15.86538462
			Saranac	1.84375	12	9.375	0.153645833	15.36458333
			USDA 96-56	1.21875	9	7.03125	0.135416667	13.54166667
			Lenape	1.9375	16	12.5	0.12109375	12.109375
			Earlaine	2.375	20	15.625	0.11875	11.875
			378971,928	1.1875	11	8.59375	0.107954545	10.79545455
			Preferent	0.96875	9	7.03125	0.107638889	10.76388889
			Furore	1	10	7.8125	0.1	10
			Katahdin	5.046875	52	40.625	0.097055288	9.705528846
4 (N=17)	146	Maternal	Gabriela	0.4375	6	35.29411765	0.072916667	7.291666667
			Katahdin	0.40625	6	35.29411765	0.067708333	6.770833333
			Saranac	0.4375	7	41.17647059	0.0625	6.25
			USDA 96.56	0.3125	6	35.29411765	0.052083333	5.208333333
			Earlaine	0.3125	6	35.29411765	0.052083333	5.208333333
			CPC 1673-20 (adg) x Furore	0.21875	5	29.41176471	0.04375	4.375
			Saskia	0.28125	7	41.17647059	0.040178571	4.017857143
	143	Paternal	Innovator	3.5	7	41.17647059	0.5	50
			RZ 84-2580	2.25	8	47.05882353	0.28125	28.125
			Katahdin	0.875	10	58.82352941	0.0875	8.75
			Saskia x (CPC 1673-20 (adg) x Furore)	0.3125	8	47.05882353	0.0390625	3.90625
			CPC 1673-11 (adg) x Furore	0.03125	1	5.882352941	0.03125	3.125
